# Endothelial progenitor cells-derived exosomal microRNA-21-5p alleviates sepsis-induced acute kidney injury by inhibiting RUNX1 expression

**DOI:** 10.1038/s41419-021-03578-y

**Published:** 2021-03-30

**Authors:** Yue Zhang, Hongdong Huang, Wenhu Liu, Sha Liu, Xue Yan Wang, Zong Li Diao, Ai Hua Zhang, Wang Guo, Xue Han, Xiaoqun Dong, Oleksandr Katilov

**Affiliations:** 1 Department of Nephrology, Beijing Friendship Hospital, Faculty of Kidney Diseases, Capital Medical University, Beijing, 100050 China; 2 Center of Allergy, Beijing Shijitan Hospital, Capital Medical University, Beijing, 100038 China; 3 Department of Internal Medicine, College of Medicine, The University of Oklahoma Health Sciences Center, Oklahoma City, OK USA; 4 Vinnitsa National Medical University, 108, Khmelnytske Highway, Vinnitsa, 21000 Ukraine

**Keywords:** Cell biology, Diseases

## Abstract

The role of microRNA-21-5p (miR-21-5p) in sepsis-induced acute kidney injury (AKI) has been seldom discussed. Therefore, the objective of this present study was to investigate the mechanism of endothelial progenitor cells-derived exosomes (EPCs-exos) in sepsis-induced AKI via miR-21-5p/runt-related transcription factor 1 (RUNX1) axis. miR-21-5p was downregulated and RUNX1 was upregulated in the kidney of cecal ligation and puncture (CLP) rats, and miR-21-5p targeted RUNX1. Elevation of miR-21-5p improved renal function and renal tissue pathological damage, attenuated serum inflammatory response, as well as reduced apoptosis and oxidative stress response in renal tissues, and regulated endothelial glycocalyx damage marker proteins syndecan-1 and heparanase-1 in CLP rats. Overexpression of RUNX1 abolished the impacts of elevated miR-21-5p in CLP rats. Also, EPCs-exos upregulated miR-21-5p expression, and functioned similar to elevation of miR-21-5p for CLP rats. Downregulating miR-21-5p partially reversed the effects of EPCs-exos on sepsis-induced AKI. Collectively, our study suggests that EPCs release miR-21-5p-containing exosomes to alleviate sepsis-induced AKI through RUNX1 silencing.

## Introduction

Sepsis is a systemic inflammatory response syndrome induced by viral, bacterial, or fungal infection, causing organ damage^1^. It is the major cause of mortality in the intensive care unit^2^. It is featured with immune inhibition, early activation of inflammatory responses, and coagulation^3^. Sepsis is one of the most common causes of acute kidney injury (AKI), accounting for ~50% of all AKI cases^4^. As a potentially deadly complication of sepsis, AKI leads to raised morbidity and mortality^5^. Albeit prompt antibiotic administration and aggressive supportive therapy, sepsis is still a life-threatening complication of infection^6^. Thus, early detection of AKI is necessary to reduce the chance of further kidney damage and to ensure a favorable outcome in septic patients.

Endothelial progenitor cells (EPCs) are widely defined as cells expressing surface markers similar to those expressed in the vascular endothelial cells that adhere to the endothelial cells and implicate in neoangiogenesis^7^. Exosomes are membrane-bound vesicles^8^ and exosomes from EPCs (EPCs-Exos) ameliorate the results of a murine model of sepsis^9^. A study has reported that EPC transplantation can dramatically decline kidney tissue damage in septic rats^10^. MicroRNAs (miRs) are a series of endogenous non-coding RNAs (18–25 nucleotides), which are capable of negatively regulating gene expression, controlling almost all cell functions including differentiation, proliferation, senescence, and apoptosis^11^. According to Pan et al.^12^, delayed remote ischemic preconditioning provides renoprotection against septic AKI via exosomal miR-21. A study has discussed the protective role of xenon on septic AKI by miR-21 target signaling pathway^13^. Runt-related transcription factor 1 (RUNX1) is recognized as indispensable for definitive hematopoiesis and is a frequent target of translocations in hematopoietic malignancies^14^. A study has discussed that RUNX1 raises toll-like receptor 4-triggered inflammation and septic shock^15^. Another study demonstrated that specific down-regulation of RUNX1 in mouse renal tubular epithelial cells diminishes renal fibrosis^16^. However, the combined effect of EPCs-Exos, miR-21-5p, and RUNX1 on sepsis-induced AKI has not been studied yet. This research aims to verify the role of EPCs-Exos, miR-21-5p, and RUNX1 in sepsis-induced AKI, and we hypothesized that EPCs-Exos and miR-21-5p may be able to alleviate sepsis-induced AKI by regulating RUNX1.

## Materials and methods

### Ethics statement

Animal experiments were strictly in accordance with the Guide to the Management and Use of Laboratory Animals issued by the National Institutes of Health. The protocol of animal experiments was approved by the Institutional Animal Care and Use Committee of Beijing Friendship Hospital, Faculty of Kidney Diseases, Capital Medical University.

### Animals

Healthy Sprague Dawley rats (aging 10–12 w and weighing 200 ± 20 g, regardless of gender) that purchased from the Experimental Animal Center at Capital Medical University (Beijing, China) were adaptively fed for 1 w in a clean grade animal house with free diet, ventilation should be carried out every 8 h.

### Culture and identification of EPCs

Rats were killed, disinfected, and placed on the super clean bench. The femur and tibia of rats were separated, the medullary cavity was rinsed by sterile D-Hank’s solution and 10 mL mixture was obtained. The mixture was added with Ficoll solution in equal proportions, then centrifuged for 10 min at 400 × *g*. The monocyte layer was absorbed and washed twice by D-Hank’s solution, suspended by α-minimum essential medium (containing 10% fetal bovine serum (FBS), 100 U/mL penicillin, and 100 U/mL streptomycin). The density of cells was set to 5 × 10^6^ cells/mL. Cells were seeded in a culture dish and cultured for 24 h, then the suspended cells were centrifuged at 100 × *g* to remove the supernatant. Next, cells were suspended by endothelial growth medium (EGM)-2MV, seeded in a cell culture dish coated with fibronectin, and cultured in a 5% CO_2_ incubator. The liquid was changed after 3 d, the non-adherent cells were removed and cells were added with fresh medium, the liquid was changed every 3 d. Cells were cultured at 37 °C, 5% CO_2_ in a humidified environment and passaged at 80–90% confluence. EPCs at passages 2–4 were used in the following experiments.

Cells were incubated with 1,1’-dioctadecyl-3,3,3′,3′-tetramethylindocarbocyanine perchlorate-acetylated-low density lipoprotein (Dil-Ac-LDL) (15 mg/L) and fluorescein isothiocyanate Ulex Europaeus agglutinin-I (FITC-UEA-I) (10 mg/L) 14 d after culture. The shuttle-type cells dyed by DiL-ac-LDL and FITC-UEA-I were observed under the inverted fluorescence microscope. The expression of surface protein CD31 and CD34 of adherent EPCs was detected by immunofluorescence assay.

miRNA antagomir NC and miR-21-5p antagomir (an antagomir is a type of specially labeled and chemically modified single-stranded miRNA, designed based on the mature miRNA sequence, which is special for inhibiting the expression of endogenous miRNA) were transfected into the EPCs by Lipofectamine 2000 (Invitrogen, CA, USA) according to the manufacturer’s instructions. The sequence of miR-21-5p antagomir were: 5′-UCAACAUCAGUCUGAUAAGCUA-3′; miRNA antagomir negative control (NC) were: 5′-CAGUACUUUUGUGUAGUACAA-3′.

### Extraction and identification of EPCs-Exos

When the confluence of EPCs reached 80%, the original medium was discarded. EPCs were treated by EGM-2MV without FBS for 1 d, then the medium was absorbed and placed in 1.5 mL Eppendorf (EP) tube, then centrifuged for 30 min at 4 °C, 2000 × *g*. The supernatant was placed in a new 1.5 mL EP tube and the precipitation was discarded. The supernatant was incubated with 1/2 volume of exosome isolation kit at 4 °C overnight. In the next day, the EP tube was centrifuged at 4 °C, 10,000 × *g* for 1 h, the precipitation was suspended by sterilized phosphate-buffered saline, the EPCs-Exos suspension was obtained and stored at −80 °C. The protein content of EPCs-Exos was gauged by bicinchoninic acid protein quantification kit. The morphological characteristics of EPCs-Exos were observed by a transmission electron microscope (TEM), exosome-specific surface proteins CD9, CD63, and CD81 were detected by western blot analysis. Exos derived from EPCs and EPCs transfected with miRNA antagomir NC or miR-21-5p antagomir were defined as Exos, Exos^antagomir NC^, and Exos^miR-21-5p antagomir^.

### Establishment of CLP rat models

Rats were starved for 12 h and weighed before operation, anesthetized by 3% pentobarbital sodium (60 mg/kg, Sigma-Aldrich Company, MO, USA)^17^ and disinfected. A 1.5 cm incision was made along the midline of the abdomen, the abdomen was opened layer by layer to expose the cecum and prevent damage to surrounding blood vessels. The surface of the cecum was wet by normal saline, the cecum was taken out and ligation was made at a distance of 1.0 cm from the cecum end. The cecum was punctured twice by no. 18 needle to squeeze out a small amount of intestinal content, and a 2.0 mm rubber band was indwelled to prevent intestinal wall closure. Then the cecum was put into the abdomen and the abdomen was sutured layer by layer. Rats were subcutaneously injected with normal saline (30 mL/kg) in the abdomen to supplement intraoperative fluid loss, then numbered and put back to the cage. After wake up, the rats could move freely and eat freely. In the sham group, the abdomen was only opened without ligation and the cecum was not punctured, then the abdomen was sutured. After 24 h of cecal ligation and puncture (CLP), rats showed signs of mental exhaustion, irritability, shivering, abdominal distension, increased secretion from the canthus, and piloerection. After the rats were killed, there were hemorrhagic exudates from the abdominal cavity, swelling, blackening, and adhesion of the cecum, and gas expanded in the intestine. There were a few intestinal and perirenal abscesses in rats, which were consistent with sepsis and could be considered a success modeling^18^.

### Grouping and treatment

Rats were divided into six groups: control group, CLP group, CLP + miRNA agomir NC (Ago-NC) group, CLP + miR-21-5p agomir (Ago-miR-21-5p) group, CLP + miR-21-5p agomir + overexpression (oe)-NC (Ago-miR-21-5p + Oe-NC) group and CLP + miR-21-5p agomir + oe-RUNX1 (Ago-miR-21-5p + Oe-RUNX1) group. The control group was established with sham-operated rats, and the CLP group was a model group. The rats in other groups were injected with miRNA agomir NC, miR-21-5p agomir (an agomir is a type of specially labeled and chemically modified double-stranded miRs, which can regulate the biological function of the target gene), miR-21-5p agomir and empty expression vector, or miR-21-5p agomir and RUNX1 overexpression vector through a tail vein before CLP operation. The sequence of miR-21-5p agomir were: 5′-UAGCUUAUCAGACUGAUGUUGA-3′; miRNA agomir negative control were: 5′-UUCUCCGAACGUGUCACGUTT-3′.

Another rats were assigned into four groups: CLP group, CLP + Exos (Exos) group, CLP + Exos^antagomir NC^ (Exos^Anta-NC^) group, CLP + Exos^miR-21-5p antagomir^ (Exos^Anta-miR-21-5p^) group. The rats of CLP, Exos, Exos^Anta-NC^, and Exos^Anta-miR-21-5p^ groups were injected with normal saline, Exos, Exos^antagomir NC^, or Exos^miR-21-5p antagomir^ through a tail vein before CLP operation.

### Sampling and treatment

Rats were anesthetized by 3% pentobarbital sodium 24 h after modeling, the blood was taken by cardiac puncture and then the rats were killed. Rats were fixed on the animal dissection table, the skin was prepared, the thorax and abdomen were opened to expose the viscera. Systemic perfusion was performed with 4% paraformaldehyde fixative solution through the heart, and the kidney was removed immediately after the color of the kidney was faded, and the right renal cortex was rapidly placed in a 1.5 mL frozen tube and preserved in liquid nitrogen. The RNA and protein was extracted for reverse transcription-quantitative polymerase chain reaction (RT-qPCR) and western blot analysis. The left kidney was cut lengthwise along the long axis and fixed with 4% paraformaldehyde for light microscope observation and immunohistochemical analysis.

### The pathological examination of rats renal tissues

The renal tissues were fixed for 1 d in 4% paraformaldehyde, dehydrated, permeabilized, and embedded with paraffin. The paraffin blocks were cooled at −20 °C, sliced on the microtome to 4 μm, then baked for 2 h at 60 °C.

#### Hematoxylin–Eosin (HE) staining

The paraffin slices were immersed in 100% xylene and a mixture of xylene and alcohol (1:1) for 15 min, dewaxed, soaked in 100%, 95%, and 75% alcohol solution for 1 min, respectively, then cleaned by 0.1 normal saline. The slices were dyed with hematoxylin for 30 min, added with 0.02% eosin and then the pathological change of renal tissues was observed under the light microscope. The score of renal tubular injury was evaluated and semi-quantitative analysis was implemented. Renal injury was defined as tubular degeneration, vacuolar degeneration, tubular type formation, tubular necrosis, and inflammatory infiltration. 0 point, normal tissues; 1 point, monoplast, focal necrosis; 2 points, the damaged area of renal tubules ≤25%; 3 points, the damaged area of renal tubules was 25–50%; 4 points, the damaged area of renal tubules was 21–75%; 5 points, the damaged area of renal tubules ≥76%.

#### Periodic acid Schiff (PAS) staining

The paraffin slices were immersed into xylene I, II, absolute ethyl alcohol I, II, 95%, 90%, 80%, and 70% alcohol, then soaked in distilled water for 2 min, oxidized by periodic acid aqueous solution for 10 min, dyed by Schiff reagent for 30 min. Then, the slices were counterstained by hematoxylin for 5 min, dehydrated by 95% alcohol and absolute ethyl alcohol, permeabilized, sealed by neutral gum, observed, and pictured under the light microscope. Determination of PAS-staining results: the nucleus was blue, the mesangial matrix, amyloid substance, and fibrin were all purplish red. The number of damaged renal tubules was counted under the light microscope.

### Determination of serum and renal tissues-related indices

The serum creatinine (Scr) and blood urea nitrogen (BUN) in serum were analyzed by HITACHI 7600-020 automatic analyzer. The levels of endothelin-1 (ET-1), inducible nitric oxide synthase (iNOS), tumor necrosis factor-α (TNF-α), and interleukin (IL)-6 in serum were tested by enzyme-linked immunosorbent assay. Scr, BUN, ET-1, iNOS, TNF-α, and IL-6 detection kits were bought from NanJing JianCheng Bioengineering Institute (Nanjing, China). The antioxidant indices in tissue homogenate were tested: superoxide dismutase (SOD) activity was measured by xanthine oxidase and malondialdeyde (MDA) was tested by thiobarbituric acid. SOD and MDA detection kits were purchased from Cayman (Ann Arbor, MI, USA).

### Terminal deoxynucleotidyl transferase-mediated deoxyuridine triphosphate-biotin nick end-labeling (TUNEL) staining

The operation was performed with reference to the TUNEL kit (Roche, Basel, Switzerland): paraffin slices were immersed in xylene I, II, absolute ethyl alcohol I, II, 95%, 90%, 80%, and 70% alcohol, then soaked in distilled water for 2 min and dripped with protease K working solution (20 μg/mL) for 30 min. The samples were incubated with TUNEL reaction mixture prepared in advance for 1 h, dried, and dripped with 50 μL converter-peroxidase for 30 min, developed by diaminobenzidine. The color development was terminated by running water. The slices were counterstained by hematoxylin, differentiated by 1% hydrochloric acid alcohol, returned to blue by running water, dehydrated, permeabilized, and sealed. The dried slices were observed under the microscope. The positive staining showed that the apoptotic cells were brown.

### RT-qPCR

The total RNA was extracted by RNA extraction kit (Invitrogen) and reversely transcribed. The prepared complementary DNA was amplified. The primers were composed by Shanghai Sangon Biotechnology Co., Ltd. (Shanghai, China) (Table 1). The ABI StepOne^TM^ real-time RT-qPCR instrument was from Applied Biosystems (Carlsbad, CA, USA). U6 was the internal reference of miR-21-5p and glyceraldehyde phosphate dehydrogenase (GAPDH) was the loading control of RUNX1. The data were reckoned by 2^−△△Ct^ method.

**Table 1:** Primer sequence.

Gene	Sequence
miR-21-5p	F: 5′-GAGATGCAGGGACACACGAT-3′
	R: 5′-ATCCGTTGATGTGCAATGCG-3′
RUNX1	F: 5′-CTGCCCATCGCTTTCAAGGT-3′
	R: 5′-GCCGAGTAGTTTTCATCATTGCC-3′
U6	F: 5′-TAAAATCTATACACGACGGCTTCG-3′
	R: 5′-TACTGTGCGTTTAAGCACTTCGC-3′
GAPDH	F: 5′-CCACTTTGTGAGCTCATTTCCTT-3′
	R: 5′-TTCGTCCTCCTCTGGTGCTCT-3′

*F* forward, *R* reverse, *miR-21-5p* microRNA-21-5p, *RUNX1* runt-related transcription factor 1, *GAPDH* glyceraldehyde phosphate dehydrogenase.

### Western blot analysis

Protein from exosomes or renal tissues was extracted using radio-immunoprecipitation assay buffer and quantified by bicinchoninic acid protein (Pierce). The protein was boiled with loading buffer at 95 °C for 5 min (40 µg/well), separated by 10% polyacrylamide gel electrophoresis, and transferred to a polyvinylidene fluoride membrane. The membrane was blocked by 5% bovine serum albumin for 1 h, added with primary antibodies CD9 (1:1000, ab92726), CD63 (1:1000, ab134045), syndecan-1 (1:1000, ab128936), heparanase-1 (1:1000, ab59787) (all from Abcam, Cambridge, MA, USA), CD81 (1:500, sc-166029, Santa Cruz Biotechnology, CA, USA), RUNX1 (1:1000, 39000, Active Motif, Carlsbad, CA, USA), and GAPDH (1:1000, Beyotime Institute of Biotechnology, Shanghai, China) at 4 °C overnight, then incubated with the corresponding secondary antibody (1:3000, MT-BIO, Shanghai, China) and developed by chemiluminescence reagent. GAPDH was used as the endogenous control. Bio-rad Gel Doc EZ imager (Bio-rad, CA, USA) was utilized for development. The gray value was analyzed by Image J software.

### Dual-luciferase reporter gene assay

The wild type (Wt) and mutant type (Mut) RUNX1-3′ untranslated region (UTR) sequences were synthesized and inserted into pGL3-basic luciferase reporter plasmid (BGI gene Co., Ltd., Guangdong, China) to form RUNX1-3′UTR-Wt and RUNX1-3′UTR-Mut. The formed vectors were combined with miR-21-5p agomir or agomir NC, and co-transfected into HEK-293T cells through Lipofectamine 2000. Cell luciferase activity was examined by Dual-Luciferase Reporter Assay System Kit (Promega, CA, USA) at Turner BioSystems (Sunnyvale, CA, USA).

### Statistical analysis

All data were indicated as the mean ± standard deviation using SPSS 20.0 for statistical analysis, and GraphPad Prism 6.0 for graph generation. Comparisons between two groups were formulated by *t* test, whereas comparisons among multiple groups were assessed by one-way analysis of variance followed by Tukey’s multiple comparisons test. *P* value < 0.05 was indicative of a statistically significant difference.

## Results

### Identification of EPCs and exosomes

EPCs cultured in vitro were observed through an inverted microscope, and it was found that the cells showed like "paving stones" when cultured to 14 d (Supplementary Fig. 1A). CD31 and CD34 in EPCs were observed by immunofluorescence staining (Supplementary Fig. 1B).

Functional identification of EPCs by FITC-UEA-I and DIL-Ac-LDL (Supplementary Fig. 1C): the fluorescence microscope showed that the cytoplasm of the EPCs showed red fluorescence, indicating that EPCs could absorb Dil-Ac-LDL; the membrane of EPCs showed green fluorescence, suggesting that EPCs could bind to FITC-UEA-I, and cells with yellow fluorescence were considered to be differentiated EPCs.

The exosomes purified by ultracentrifugation were counterstained with 3% phosphotungstic acid. Under the TEM, exosomes were presented with a complete bilayer vesicle structure with 30-120 nm diameter, and the size was conformed to the general features of exosomes (Supplementary Fig. 1D).

The expression of exosomes specific marker proteins CD9, CD63, and CD81 was tested by western blot analysis, it was reported that CD9, CD63, and CD81 could be detected (Supplementary Fig. 1E). It was confirmed that exosomes were successfully isolated.

miR-21-5p expression in Exos derived from EPCs transfected with miRNA antagomir NC (Exos^antagomir NC^) or miR-21-5p antagomir (Exos^miR-21-5p antagomir^) was determined by RT-qPCR. The results reflected that (Supplementary Fig. 1F), versus the Exos^antagomir NC^ group, decreased miR-21-5p was found in the Exos^miR-21-5p antagomir^ group (*P* < 0.05).

### Effects of upregulation of miR-21-5p on renal function and pathological changes in the kidney of CLP rats

**Fig. 1:**
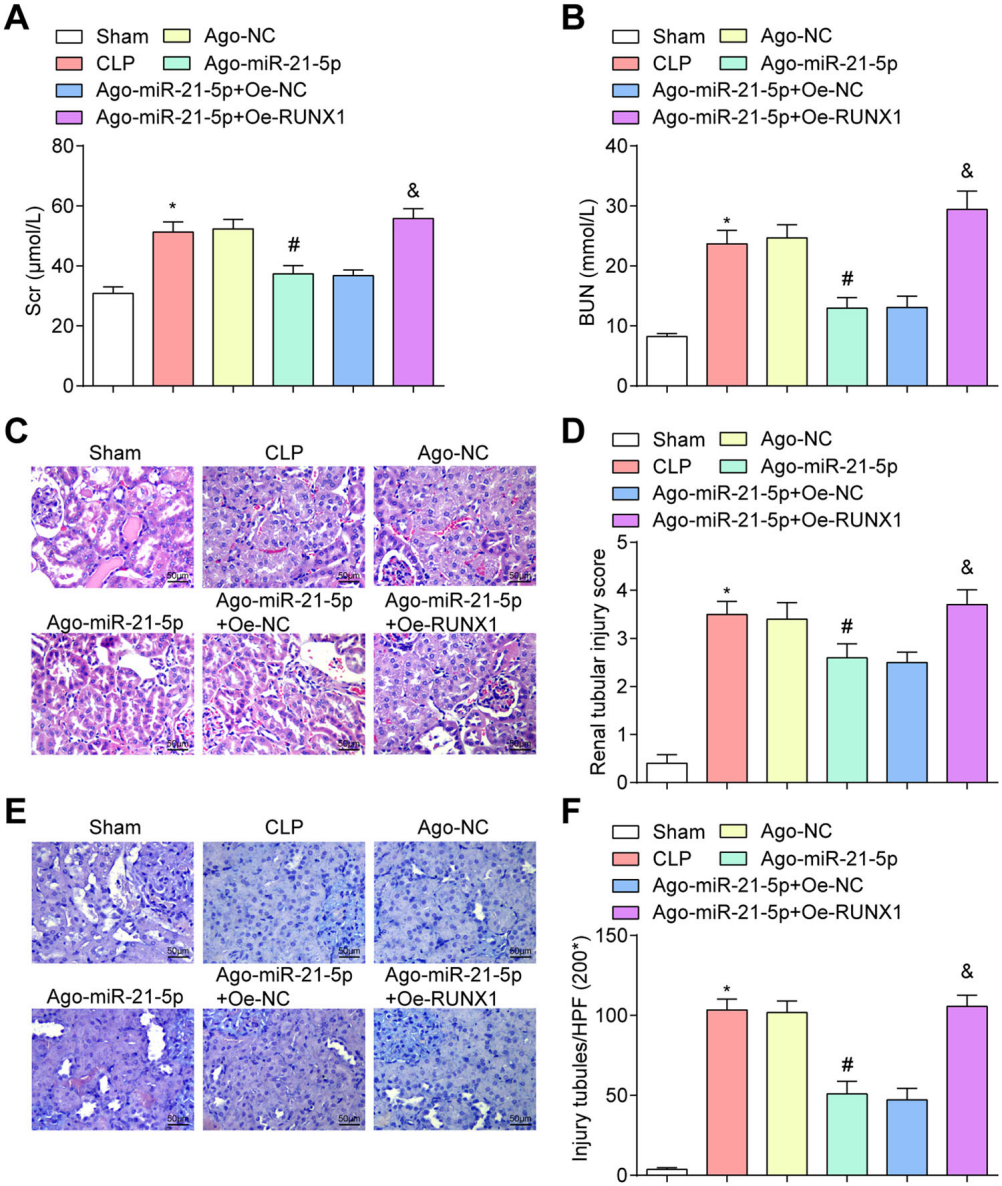
Effects of upregulation of miR-21-5p on renal function and pathological changes in the kidney of CLP rats. **A** Comparison of Scr concentration in CLP rats. **B** Comparison of BUN concentration in CLP rats. **C** HE staining of rat renal tissues (scale bar = 50 μm). **D** Comparison of renal tubular injury scores in CLP rats. **E** PAS staining of rat renal tissues of CLP rats (scale bar = 50 μm). **F** Comparison of the number of renal tubular injuries of CLP rats. *n* = 10. *P < 0.05 vs. the sham group. ^#^*P* < 0.05 vs. the Ago-NC group. ^&^*P* < 0.05 vs. the Ago-miR-21-5p + Oe-NC group. Measurement data were depicted as mean ± standard deviation.

Versus the sham group and the Ago-miR-21-5p + Oe-NC group, Scr and BUN levels were raised in the CLP group and the Ago-miR-21-5p + Oe-RUNX1 group (all *P* < 0.05). In relation to the Ago-NC group, Scr and BUN levels were decreased in the Ago-miR-21-5p group (all *P* < 0.05) (Fig. 1A, B).

HE staining results reported that the renal tubules in the sham group were basically normal. In the CLP group, there was obvious tubular necrosis, hyaline cast, accompanied by a large number of mononuclear infiltration. The rats in the Ago-miR-21-5p group and Ago-miR-21-5p + Oe-NC group exhibited reduced renal tubular vacuolar degeneration, and significantly reduced monocyte infiltration. The situations of rats in the Ago-NC group and the Ago-miR-21-5p + Oe-RUNX1 group were the same as those in the CLP group. Compared with the sham group, the score of tubular injury was heightened in the CLP group (*P* < 0.05). Versus the Ago-NC group, the score of tubular injury was depressed, the denaturation of renal tubules, mononuclear infiltration, and monocyte infiltration was reduced in the Ago-miR-21-5p group (*P* < 0.05). In comparison to the Ago-miR-21-5p + Oe-NC group, the score of tubular injury was heightened in the Ago-miR-21-5p + Oe-RUNX1 group (*P* < 0.05) (Fig. 1C, D).

PAS staining revealed that the structure of renal tissues was normal in the sham group. In the CLP group, some renal tubules were dilated and epithelial cells with disintegration and necrosis were exfoliated, such as lumen, and presented granular structure. There were swelling, necrosis, and vacuolar degeneration of renal tubular epithelial cells, the disappearance of brush border, necrosis, and disintegration of some tubular epithelial cells, infiltration of inflammatory cells in the renal stroma, increased tubular shape, and aggravation of the renal injury. The renal injury in the Ago-miR-21-5p group was mitigated versus the Ago-NC group, and the renal tubular epithelial cell necrosis was alleviated, a discontinuous brush border could be seen and the renal cast was reduced. The renal injury of rats in the Ago-miR-21-5p + Oe-RUNX1 group was remarkably worse than that in the Ago-miR-21-5p + Oe-NC group, and the renal cast was increased (all *P* < 0.05) (Fig. 1E, F).

### Highly expressed miR-21-5p increases syndecan-1 and reduces heparanase-1 expression in renal tissues of CLP rats

**Fig. 2:**
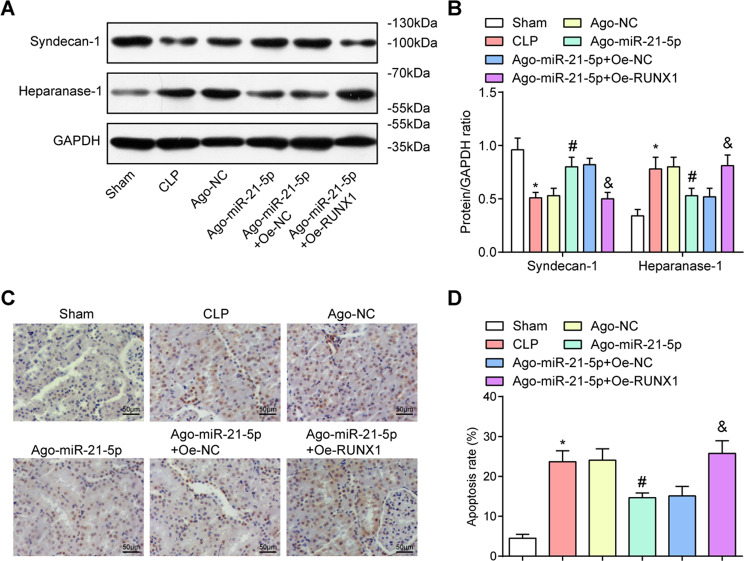
Highly expressed miR-21-5p increases syndecan-1 and reduces heparanase-1 expression and apoptosis in renal tissues of CLP rats. **A** Protein bands of syndecan-1 and heparanase-1 in renal tissues of rats. **B** Comparison of syndecan-1 and heparanase-1 protein contents in renal tissues of rats. **C** TUNEL staining was used to detect the apoptosis of rat renal cells (scale bar = 50 μm). **D** Comparison of renal cell apoptosis rate in CLP rats. *n* = 10. *P < 0.05 vs. the sham group. ^#^*P* < 0.05 vs. the Ago-NC group. ^&^*P* < 0.05 vs. the Ago-miR-21-5p + Oe-NC group. Measurement data were depicted as mean ± standard deviation.

Syndecan-1 has correlated with baseline endothelial dysfunction and the skeleton of endothelial glycocalyx^19–21^. Heparanase-1 is activated in AKI and has an important role in endothelial glycocalyx and shedding^22,23^. Syndecan-1 and heparanase-1 protein contents were tested by western blot analysis (Fig. 2A, B) compared with the sham group and the Ago-miR-21-5p + Oe-NC group, syndecan-1 protein level declined while heparanase-1 protein level was enhanced in the CLP group and the Ago-miR-21-5p + Oe-RUNX1 group (all *P* < 0.05). Versus the Ago-NC group, syndecan-1 protein level was raised while heparanase-1 protein level was reduced in the Ago-miR-21-5p group (both *P* < 0.05).

### Overexpression of miR-21-5p reduces apoptosis in renal tissues of CLP rats

The apoptotic cells were detected by TUNEL staining, and the outcomes revealed that in the sham group, there were almost no apoptotic cells. In the CLP group, the Ago-NC group, and the Ago-miR-21-5p + Oe-RUNX1 group, there were clusters and queues of apoptotic cells. In the Ago-miR-21-5p group and the Ago-miR-21-5p + Oe-NC group, scattered apoptotic cells could be seen, indicating that upregulation of miR-21-5p could reduce TUNEL-positive cells in renal tissues of CLP rats. Versus the sham group and the Ago-miR-21-5p + Oe-NC group, the TUNEL-positive cells were increased in the CLP group and the Ago-miR-21-5p + Oe-RUNX1 group (both *P* < 0.05). In comparison with the Ago-NC group, the TUNEL-positive cells were decreased in the Ago-miR-21-5p group (*P* < 0.05) (Fig. 2C, D).

### Restored miR-21-5p attenuates the inflammatory and oxidative stress responses in CLP rats

**Fig. 3:**
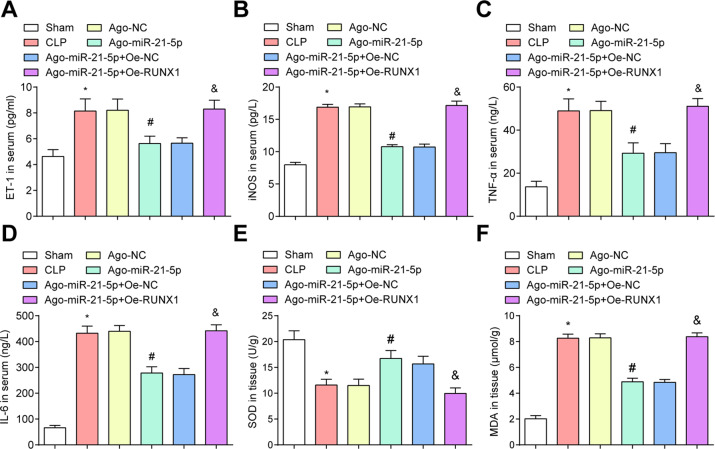
Restored miR-21-5p attenuates the levels of serum-related indices and oxidative stress response in CLP rats. **A** Comparison of serum ET-1 levels in CLP rats. **B** Comparison of serum iNOS levels in CLP rats. **C** Comparison of serum TNF-α levels in CLP rats. **D** Comparison of serum IL-6 levels in rats. **E** Comparison of SOD activity in CLP rats. **F** Comparison of tissue MDA levels in CLP rats. *n* = 10. *P < 0.05 vs. the sham group. ^#^*P* < 0.05 vs. the Ago-NC group. ^&^*P* < 0.05 vs. the Ago-miR-21-5p + Oe-NC group. Measurement data were depicted as mean ± standard deviation.

Serum-related indices presented that in relation to the sham group and the Ago-miR-21-5p + Oe-NC group, ET-1, iNOS, TNF-α, and IL-6 levels were enhanced in the CLP group and the Ago-miR-21-5p + Oe-RUNX1 group (all *P* < 0.05). Versus the Ago-NC group, ET-1, iNOS, TNF-α, IL-6 levels were diminished in the Ago-miR-21-5p group (all *P* < 0.05) (Fig. 3A–D).

The oxidative stress response in renal tissues of rats was measured and the finding indicated that MDA levels were raised while SOD activity was reduced in the CLP group and the Ago-miR-21-5p + Oe-RUNX1 group (all *P* < 0.05). Versus the Ago-NC group, MDA levels were diminished while SOD activity was enhanced in the Ago-miR-21-5p group (both *P* < 0.05) (Fig. 3E, F).

### miR-21-5p expression is reduced while RUNX1 expression is raised in renal tissues of CLP rats, exosomes raise miR-21-5p expression in renal tissues of CLP rats and miR-21-5p targets RUNX1

**Fig. 4:**
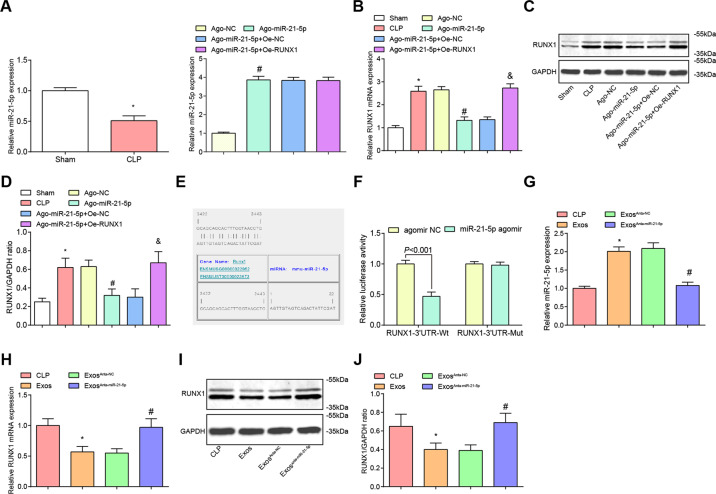
miR-21-5p expression is reduced while RUNX1 expression is raised in renal tissues of CLP rats, exosomes raise miR-21-5p expression in renal tissues of CLP rats and miR-21-5p targets RUNX1. **A**/**G** The expression of miR-21-5p in renal tissues of CLP rats detected by RT-qPCR. **B**/**H** The expression of RUNX1 mRNA in renal tissues of CLP rats detected by RT-qPCR. **C**/**I** Protein band of RUNX1 in renal tissues of CLP rats. **D**/**J** The expression of RUNX1 protein in renal tissues of rats detected by western blot analysis. **E** The binding site of miR-21-5p and RUNX1 predicted by online software. **F** The target relationship between miR-21-5p and RUNX1 verified by dual-luciferase reporter gene assay. **A**–**D**, *n* = 10. *P < 0.05 vs. the sham group. ^#^*P* < 0.05 vs. the Ago-NC group. ^&^*P* < 0.05 vs. the Ago-miR-21-5p + Oe-NC group. **G**–**J** *n* = 10. *P < 0.05 vs. the CLP group. ^#^*P* < 0.05 vs. the Exos^Anta-NC^ group. **F** *N* = 3. Measurement data were depicted as mean ± standard deviation.

RT-qPCR and western blot analysis presented that versus the sham group, miR-21-5p expression was declined while RUNX1 expression was heightened in renal tissues in the CLP group. By comparison with the Ago-NC group, miR-21-5p expression was enhanced while RUNX1 expression was reduced in the Ago-miR-21-5p group (both *P* < 0.05). The expression trend of miR-21-5p was opposite to that of RUNX1, which further indicated that there might be an interaction between the two. In relation to the Ago-miR-21-5p + Oe-NC group, RUNX1 expression was elevated in the Ago-miR-21-5p + Oe-RUNX1 group (*P* < 0.05) (Fig. 4A–D).

The target relationship between RUNX1 and miR-21-5p was predicated by bioinformatics software (https://cm.jefferson.edu/rna22/Precomputed) (Fig. 4E). The results of dual-luciferase reporter gene assay revealed that (Fig. 4F) the luciferase activity of RUNX1-3′UTR-Wt was reduced by the co-transfection with miR-21-5p agomir (*P* < 0.05), whereas that of RUNX1-3′UTR-Mut was not altered in response to the co-transfection of miR-21-5p agomir (*P* > 0.05).

Compared with the CLP group, miR-21-5p expression was enhanced while RUNX1 expression was reduced in the Exos group (both *P* < 0.05). By comparison with the Exos^Anta-NC^ group, miR-21-5p expression was reduced while RUNX1 expression was elevated in the Exos^Anta-miR-21-5p^ group (both *P* < 0.05) (Fig. 4G–J).

### Effects of EPCs-Exos on renal function and pathological changes in the kidney of CLP rats

**Fig. 5:**
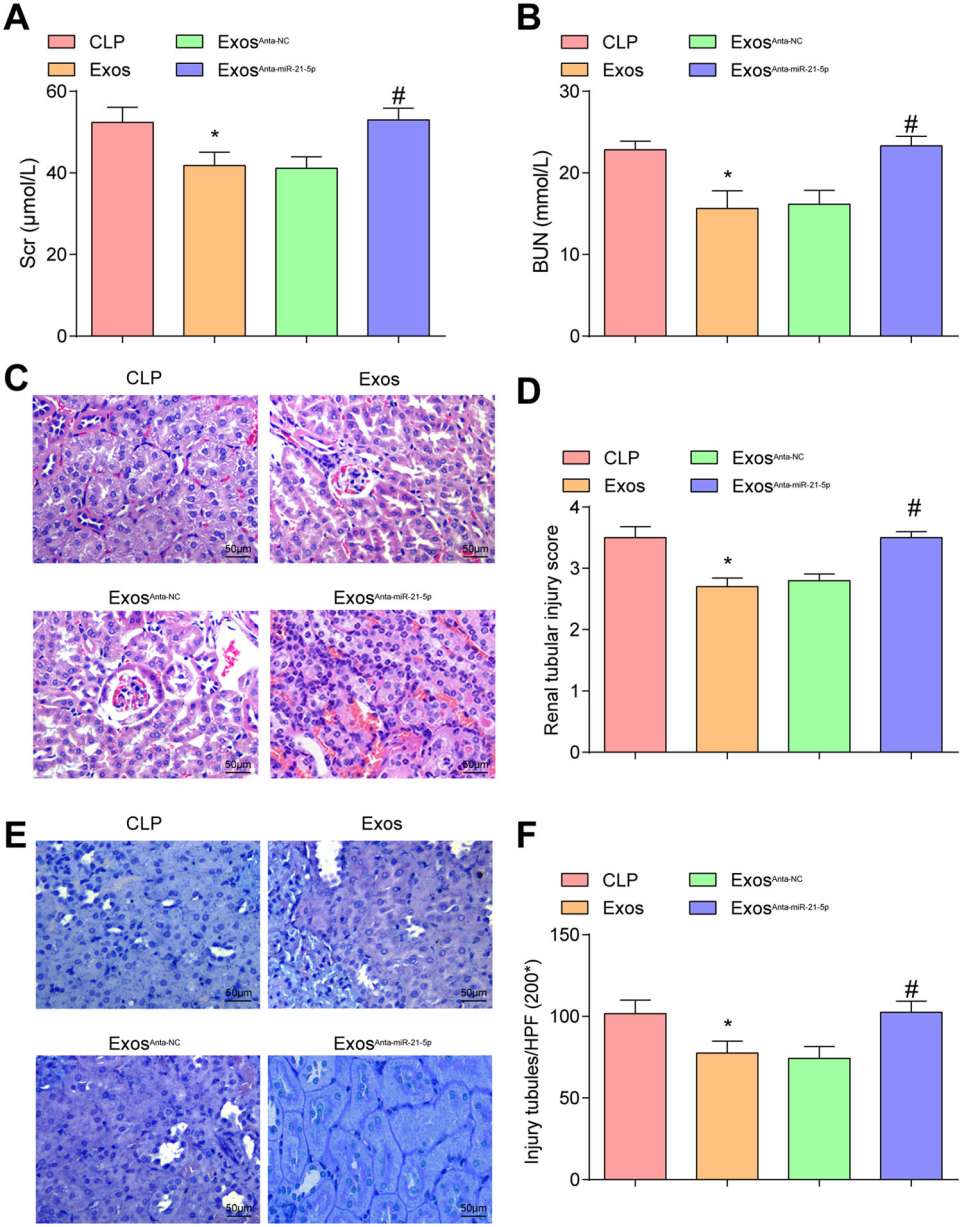
Effects of EPCs-Exos on renal function and pathological changes in the kidney of CLP rats. **A** Comparison of Scr concentration in CLP rats. **B** Comparison of BUN concentration in CLP rats. **C** HE staining of rat renal tissues of CLP rats (scale bar = 50 μm). **D** Comparison of renal tubular injury scores. **E** PAS staining of rat renal tissues of CLP rats (scale bar = 50 μm). **F** Comparison of the number of renal tubular injuries of CLP rats. *n* = 10. *P < 0.05 vs. the CLP group. ^#^*P* < 0.05 vs. the Exos^Anta-NC^ group. Measurement data were depicted as mean ± standard deviation.

The results of renal index detection reported that (Fig. 5A, B) in contrast with the CLP group, Scr and BUN levels were depressed in the Exos group (both *P* < 0.05). Versus the Exos^Anta-NC^ group, Scr and BUN levels were raised in the Exos^Anta-miR-21-5p^ group (both *P* < 0.05).

HE staining results displayed that (Fig. 5C, D) CLP group showed clear tubular necrosis and hyaline cast with a large number of mononuclear infiltration. In contrast with the CLP group, the Exos group showed reduced tubular vacuolar degeneration, mononuclear cell infiltration, and tubular damage score (*P* < 0.05). Versus the Exos^Anta-NC^ group, the tubular damage score was raised in the Exos^Anta-miR-21-5p^ group (*P* < 0.05).

It was suggested by PAS staining that (Fig. 5E, F) in the CLP group, there were histopathological changes, loss of brush border, tubular degeneration, and cortical tubular degeneration. In contrast with the CLP group, the Exos group showed obvious improvement in renal injury (*P* < 0.05), with less swelling, necrosis, and exfoliation of renal tubular epithelial cells, and less renal cast. Versus the Exos^Anta-NC^ group, the renal injury was aggravated with loss of brush border and accentuated renal cast in the Exos^Anta-miR-21-5p^ group (*P* < 0.05).

### EPCs-Exos raise syndecan-1 and reduce heparanase-1 levels in renal tissues of CLP rats

**Fig. 6:**
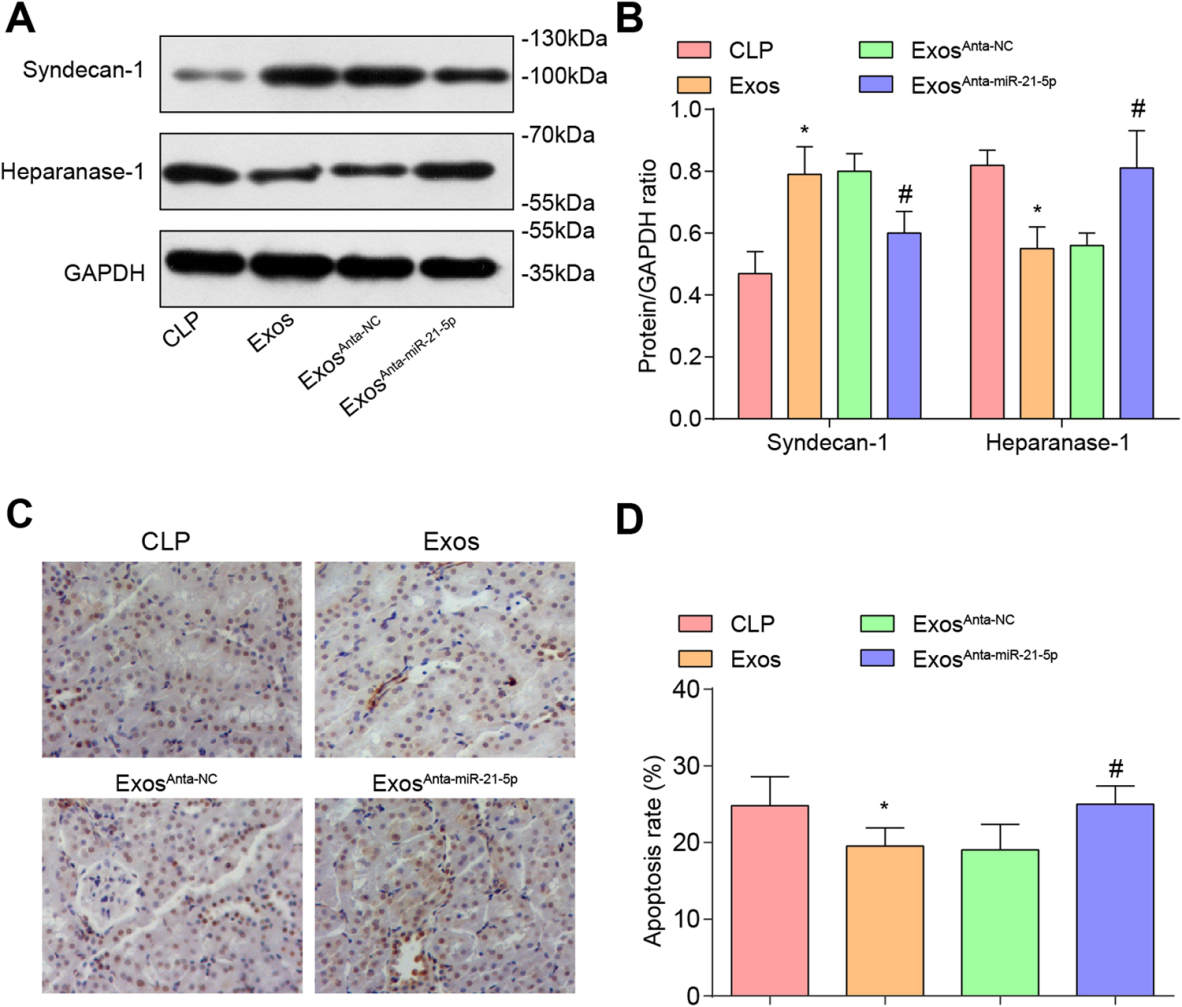
EPCs-Exos raise syndecan-1 and reduce heparanase-1 levels, and reduce apoptosis in renal tissues of CLP rats. **A** Protein bands of syndecan-1 and heparanase-1 in renal tissues of CLP rats. **B** Comparison of syndecan-1 and heparanase-1 protein contents in renal tissues of CLP rats. **C** TUNEL staining was used to detect the apoptosis of rat renal cells (scale bar = 50 μm). **D** Comparison of renal cell apoptosis rate in CLP rats. *n* = 10. *P < 0.05 vs. the CLP group. ^#^*P* < 0.05 vs. the Exos^Anta-NC^ group. Measurement data were depicted as mean ± standard deviation.

Western blot analysis was utilized to detect syndecan-1 and heparanase-1 protein contents, it was reported that (Fig. 6A, B) compared with the CLP group, syndecan-1 protein content was enhanced while heparanase-1 protein content was declined in the Exos group (both *P* < 0.05). Versus the Exos^Anta-NC^ group, syndecan-1 protein content was reduced while heparanase-1 protein content was elevated in the Exos^Anta-miR-21-5p^ group (both *P* < 0.05).

### EPCs-Exos reduce apoptosis in renal tissues of CLP rats

TUNEL staining results revealed that in the CLP group, there were clusters and queues of apoptotic cells. In the Exos group and the Exos^Anta-NC^ group, scattered apoptotic cells could be seen, suggesting that exosomes treatment could reduce TUNEL-positive cells. Versus the CLP group, the TUNEL-positive cells were markedly reduced in the Exos group (*P* < 0.05). In comparison to the Exos^Anta-NC^ group, the TUNEL-positive cells were increased in the Exos^Anta-miR-21-5p^ group (*P* < 0.05) (Fig. 6C, D).

### EPCs-Exos diminish the inflammatory and oxidative stress responses in CLP rats

**Fig. 7:**
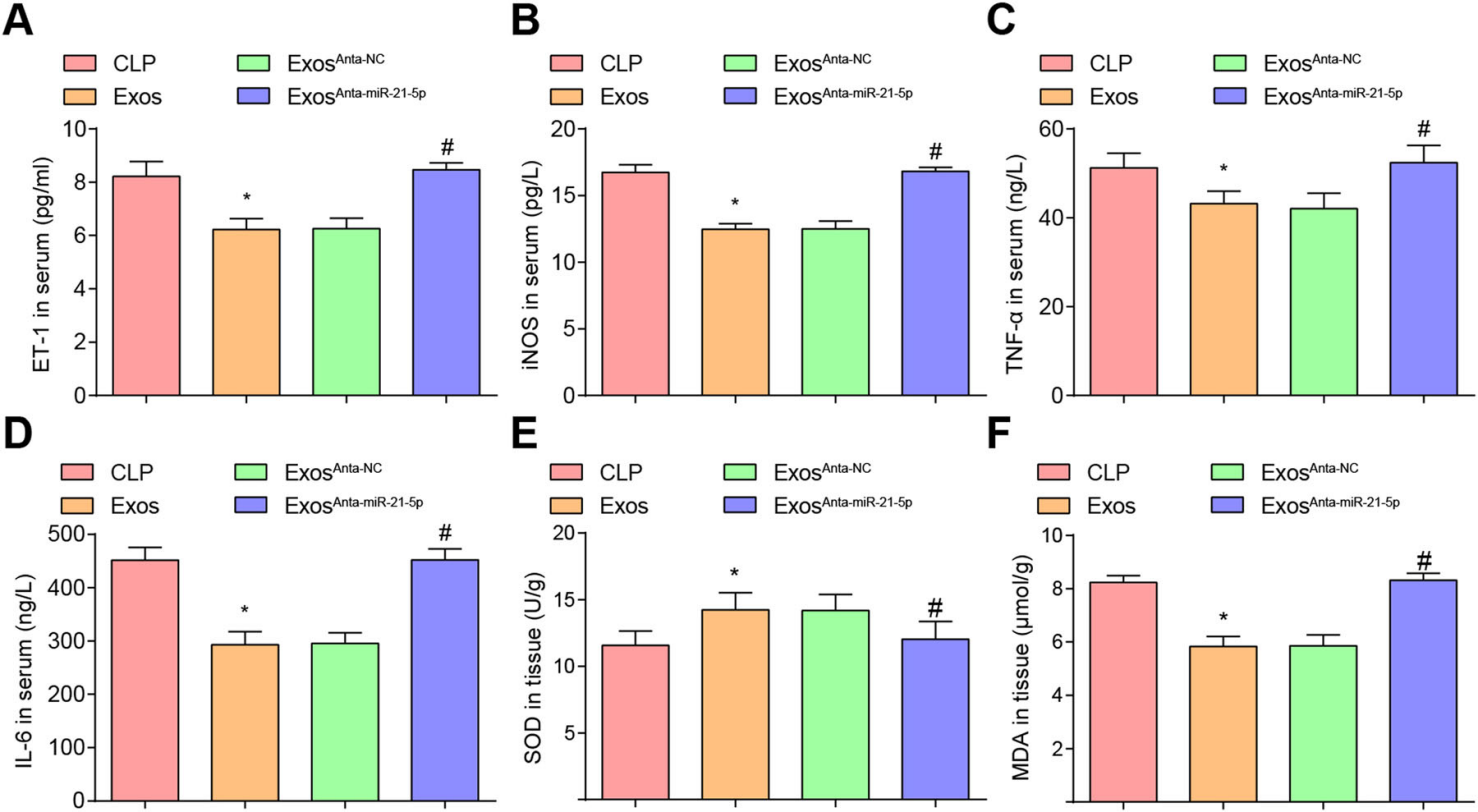
EPCs-Exos diminish the inflammatory and oxidative stress responses in CLP rats. **A** Comparison of serum ET-1 levels in CLP rats. **B** Comparison of serum iNOS levels in CLP rats. **C** Comparison of serum TNF-α levels in CLP rats. **D** Comparison of serum IL-6 levels in CLP rats. **E** Comparison of tissue SOD activity in CLP rats. **F** Comparison of tissue MDA levels in CLP rats. *n* = 10. *P < 0.05 vs. the CLP group. ^#^*P* < 0.05 vs. the Exos^Anta-N^^C^ group. Measurement data were depicted as mean ± standard deviation.

The results of serum-related indices displayed that versus the CLP group, ET-1, iNOS, TNF-α, and IL-6 levels were suppressed in the Exos group (all *P* < 0.05). By comparison with the Exos^Anta-NC^ group, ET-1, iNOS, TNF-α, and IL-6 levels were enhanced in the Exos^Anta-miR-21-5p^ group (all *P* < 0.05) (Fig. 7A–D).

MDA level and SOD activity were also measured in renal tissues of rats. It was observed that MDA level was reduced and SOD activity was strengthened in the Exos group (both *P* < 0.05). Versus the Exos^Anta-NC^ group, the MDA level was elevated and SOD activity was impaired in the Exos^Anta-miR-21-5p^ group (both *P* < 0.05) (Fig. 7E, F).

## Discussion

Sepsis-induced AKI is a serious complication of sepsis and the prevailing cause of death in intensive care unit patients^24^. In a study conducted by Zhou et al.^9^, it is shown that EPCs-exosomes can ameliorate sepsis outcomes potentially and prevent microvascular dysfunction. A study has suggested that exosomal miR-21 is implicated in renoprotection in sepsis-induced AKI^12^. It is customarily considered that RUNX1 can be a new potential target for preventing sepsis^15^. We aim to explore the effect of EPCs-Exos delivering miR-93 on sepsis-induced AKI by mediating RUNX1.

Our results reported that RUNX1 expression was raised and miR-21-5p expression was declined in renal tissues. A study has presented that miR-21 is dramatically reduced in the kidneys of septic AKI^12^. Another study has presented that miR-21 expression is declined in kidney of septic AKI^13^. A study has purported that the expression of RUNX1 is markedly enhanced in renal fibrosis^16^. It has been demonstrated that RUNX1 expression is notably heightened in the nuclei of renal epithelial cells, also in human autosomal dominant polycystic kidney disease samples^25^ It is reported that miR-21-5p is highly enriched in EPCs-exosomes^26^. It was reported by our study that exosomes raised miR-21-5p and decreased RUNX1 in renal tissues. A study has purported that exosomal miR-21-5p is enhanced in the serum of papillary thyroid cancer patients^27^. The relation between exosomes and miR-21-5p or RUNX1 in renal tissues needs further study. Moreover, a result that emerges from our study was that miR-21-5p directly targeted RUNX1. Some studies have revealed the target relation between miRs and RUNX1, including that miR-141 induces cell apoptosis via targeting of RUNX1 in prostatic cancer^28^ and RUNX1 was targeted by miR-18a-5p in malignant melanoma^29^. Nevertheless, the target relation between miR-21-5p and RUNX1 in sepsis-induced AKI has not been uncovered before.

In addition, our data revealed that exosomes or overexpression of miR-21-5p reduced Scr and BUN levels, renal tubular injury score, apoptosis rate, inflammatory factors levels, and oxidative stress response as well as increased the proportion of endothelial glycocalyx area in glomerulus in sepsis-induced AKI. A study shows that Scr and BUN levels, apoptotic cell scores, TNF-α and IL-1β levels, and oxidative stress are raised as well as survival rates and renal histology scores are declined in the sepsis-induced AKI^30^. It is indicated in a study that SOD activity is decreased and MDA level, BUN, and the pathological damage are increased in kidney tissue of sepsis-induced kidney injury^31^. According to Dogné and Flamion, the endothelial glycocalyx is damaged in acute injury, inflammatory conditions, or many other pathologic conditions^32^. The oxidative stress and inflammatory, and apoptosis of tubular cell and tubular necrosis are reported to be raised in the sepsis-induced AKI mice model^33^. It has been suggested previously that there is a reduce in BUN and Cr levels, apoptosis, necrosis of proximal kidney tubules, and oxidative stress in AKI rats treated with exosomes, moreover, exosomes attenuates the number of apoptotic cells, oxidative stress, and activation in vitro^34^. It is reported that exosomes treatment markedly restrains the Scr and BUN levels, alleviates the morphological damage and suppresses renal tubular cells apoptosis in sepsis-associated AKI^35^. A study has verified that mice receiving anti-miR-21 treatment shows a raise of apoptotic cells in kidneys^13^. It is displayed that downregulating miR-21 remarkably reduces the anti-apoptotic effects with increased number of apoptotic cells in septic AKI^12^.

Together, our findings identify that EPCs-derived exosomal miR-21-5p can alleviate the kidney injury caused by sepsis by downregulating RUNX1, thus achieving the endothelial protection of kidney tissues in sepsis-induced AKI. Owing to the limited known researches, the exact mechanism of miR-21-5p and RUNX1 is not fully elucidated, and therefore, further studies are required to illustrate the underlying mechanism.

## Supplementary information

Supplement figure 1 legend

Supplementary Figure 1
