# Supplementary material for: Endothelial progenitor cells-derived exosomal microRNA-21-5p alleviates sepsis-induced acute kidney injury by inhibiting RUNX1 expression
Source: Cell Death Dis. 2021 Mar 30;12(4):335. doi: 10.1038/s41419-021-03578-y (PMC8009943; doi:10.1038/s41419-021-03578-y)
Supplement: Supplementary file 1 — Supplement figure 1 legend [file 41419_2021_3578_MOESM1_ESM.doc]

**Supplementary Figure 1** Identification of EPCs and exosomes. A, Primary EPCs were observed. B, CD31 and CD34 staining of EPCs. C, EPCs combined with FITC-UEA-I, absorbed DIL-Ac-LDL, nucleus staining by DAPI and image fusion. D, Morphology of exosomes under TEM (scale bar: 100 nm). E, Exosome surface marker proteins CD9, CD63, and CD81 tested by western blot analysis. F. miR-21-5p expression in Exos derived from EPCs transfected with miRNA antagomir NC (Exosantagomir NC) or miR-21-5p antagomir (ExosmiR-21-5p antagomir) tested by RT-qPCR.
