# Supplementary figures and images for: Endothelial progenitor cells-derived exosomal microRNA-21-5p alleviates sepsis-induced acute kidney injury by inhibiting RUNX1 expression
Source: Cell Death Dis. 2021 Mar 30;12(4):335. doi: 10.1038/s41419-021-03578-y (PMC8009943; doi:10.1038/s41419-021-03578-y)

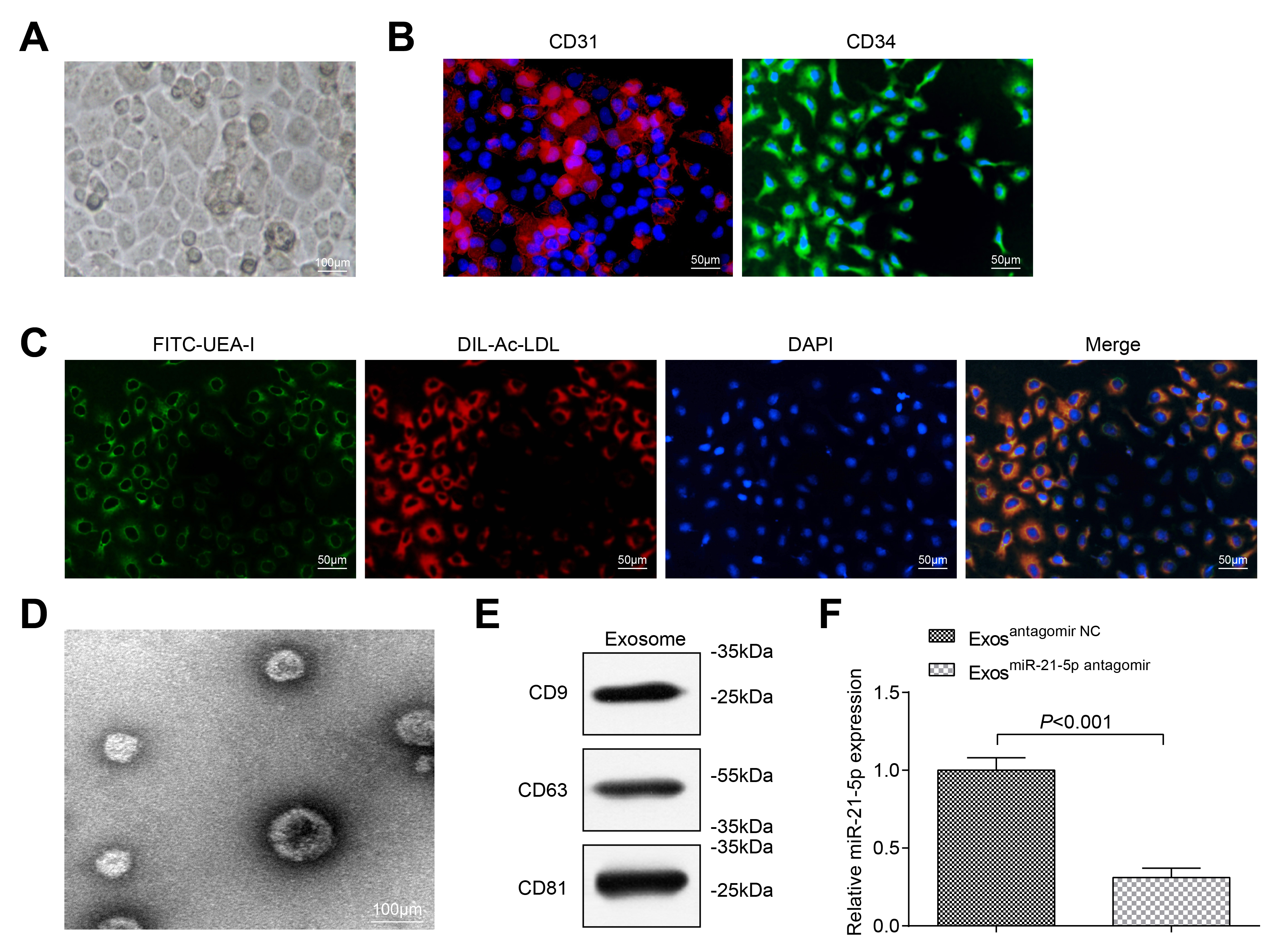

Supplement: Supplementary file 2 — Supplementary Figure 1 [file 41419_2021_3578_MOESM2_ESM.jpg]
